# Purification and Characterization of a Novel Laccase from *Cerrena* sp. HYB07 with Dye Decolorizing Ability

**DOI:** 10.1371/journal.pone.0110834

**Published:** 2014-10-30

**Authors:** Jie Yang, Qi Lin, Tzi Bun Ng, Xiuyun Ye, Juan Lin

**Affiliations:** 1 College of Biological Sciences and Technology, Fuzhou University, Fuzhou, Fujian, China; 2 National Engineering Laboratory for Enzyme Expression, Fuzhou, Fujian, China; 3 School of Biomedical Sciences, Faculty of Medicine, The Chinese University of Hong Kong, Shatin, New Territories, Hong Kong, China; Tecnologico de Monterrey, Mexico

## Abstract

Laccases (EC 1.10.3.2) are a class of multi-copper oxidases with important industrial values. A basidiomycete strain *Cerrena* sp. HYB07 with high laccase yield was identified. After cultivation in the shaking flask for 4 days, a maximal activity of 210.8 U mL^−1^ was attained. A 58.6-kDa laccase (LacA) with 7.2% carbohydrate and a specific activity of 1952.4 U mg^−1^ was purified. 2,2′-Azino-bis (3-ethylbenzothiazoline-6-sulfonic acid) was the optimal substrate, with *K*_m_ and *k*_cat_ being 93.4 µM and 2468.0 s^−1^, respectively. LacA was stable at 60°C, pH 5.0 and above, and in organic solvents. Metal ions Na^+^, K^+^, Ca^2+^, Mg^2+^, Mn^2+^, Zn^2+^ enhanced LacA activity, while Fe^2+^ and Li^+^ inhibited LacA activity. LacA decolorized structurally different dyes and a real textile effluent. Its gene and cDNA sequences were obtained. Putative *cis*-acting transcriptional response elements were identified in the promoter region. The high production yield and activity, robustness and dye decolorizing capacity make LacA and *Cerrena* sp. HYB07 potentially useful for industrial and environmental applications such as textile finishing and wastewater treatment.

## Introduction

Dyestuffs are lost in industrial effluents, posing health and environmental threats [1], [2]. Traditional processes cannot remove all dyes or are costly [3]. Microbial treatments suggested for degradation of azo and triphenylmethane dyes often result in mutagenic or carcinogenic products under anaerobic conditions [1], [4]. In contrast, laccases may provide a green and efficient alternative for decolorizing dyes before discharge into sewage treatment systems or the environment. By oxidation, laccases catalyze dye transformation, leading to dye decolorization and often detoxification as well [3]–[7].

Laccases are copper-containing oxidases catalyzing oxidation of phenolic/non-phenolic lignin-related compounds and recalcitrant environmental pollutants [8]. Artificial or natural redox mediators such as 2,2′-azino-bis(3-ethylbenzothiazoline-6-sulfonic acid) (ABTS) and acetosyringone (ACE) broaden the range of laccase substrates [4]. Because laccases have low substrate specificity, utilize oxygen as final electron acceptor and produce water as only by-product, they find applications in paper pulping and bleaching, textile refining, dye decolorization, bioremediation, organic synthesis, juice and wine clarification, etc. [3].

White-rot fungi are the most efficient laccase producers and most intensively studied. Proposed roles of fungal laccases include morphogenesis, plant pathogenesis, pigment production and lignin degradation [8].

Although laccases have been studied for decades, their applications are hampered by low production yields and reduced performance under industrial conditions. Work is under way to explore sources of laccases with easy availability, high catalytic efficiency, broad substrate specificity, tolerance to alkaline conditions, high temperatures and organic solvents, etc. [5], [9]–[11] *Pleurotus ostreatus* and *Trametes versicolor* are two model organisms in laccase research [12], [13]. Other fungi, such as those from the genera *Cerrena* and *Ganoderma*, may still provide laccases with remarkable qualities [5], [9].

To date, publications on laccase-manufacturing *Cerrena* species mainly dealt with their isolation from the environment, fermentation medium and condition optimization, purification and biochemical characterization of laccases and potential applications in bioremediation and biodegradation [9], [14]–[20]. In the present work, a novel *Cerrena* sp. strain HYB07 with strong laccase activity was identified. A laccase, designated as LacA, was purified from the fermentation broth of HYB07. The biochemical characteristics, kinetic properties and dye/effluent decolorizing potentials of LacA were investigated, and the gene and cDNA sequences of *LacA* were cloned. The research presented herein provides a novel laccase with high yield and specific activity, thermo- and pH-stability, wide substrate range and strong dye decolorizing ability, which are important for industrial processes such as biodegradation and bioremediation.

## Materials and Methods

### 2.1 Organisms and media

The strain *Cerrena* sp. HYB07 [21] was kept in the culture collection of College of Biological Sciences and Technology, Fuzhou University and maintained through periodic transfers on potato dextrose agar (PDA) (Difco, Franklin Lakes, New Jersey, USA) at 4°C. For laccase fermentation, five mycelial plugs (1 cm diameter) were removed from the peripheral region of 3-d-old PDA plates and inoculated in potato dextrose broth (PDB) (Difco, Franklin Lakes, New Jersey, USA). After growing for 3 d at 28°C and 150 rpm, an aliquot was taken to inoculate PDB medium supplemented with 0.5% yeast extract (PDY) and 0.4 mM CuSO_4_.

### 2.2 Phylogenetic analysis

Phylogeny of the strain was identified by 18S rDNA sequencing. Genomic DNA was extracted with a DNA Quick Plant System (TIANGEN, Beijing, China), and universal primers NS1 and NS8 (Table 1) were used for amplification of 18S rDNA [22]. The PCR product was ligated with the pMD18-T vector (Takara, Dalian, China), and the ligation products were transformed into *E. coli* TOP10 competent cells (Life Technologies, Grand Island, New York, USA). Four individual clones were sequenced. The 18S rDNA sequence has been submitted to GenBank with the accession number KM233493. The phylogenetic analysis (with 1,000 bootstraps) was performed with MEGA version 5.0 (http://www.megasoftware.net/) by the neighbor-joining method. Other fungal 18S rDNA sequences used in this study were from GenBank.

**Table 1:** Primers used in this study.

Primer	Oligonucleotide sequence	Reference
NS1	5′-GTAGTCATATGCTTGTCTC-3′	[22] a
NS8	5′-TCCGCAGGTTCACCTACGGA-3′	
Cu I	5′-CAYTGGCAYGGNTTYTTYCA-3′	[29] b
Cu IV	5′-TGVHARTCDATRTGRCARTG-3′	
LacA SP1	5′- CGACATCATAGAGAGCCTTGTGCGGA -3′	This study^c^
LacA SP2	5′- TCAGTGGGATCGTAGACAACAAAGGCA -3′	
LacA SP3	5′- AGATGGGGCACTGGTTCACGA -3′	
LacA SP4	5′- TCTGGCTCCGTCATTACCCTTCCCATC -3′	
LacA SP5	5′- AGACCATTGAACTTACCCTTGCAGCG -3′	
LacA SP6	5′- GGTCAAACTACTCCCAACTACG -3′	
AD1	5′- TGWGNAGWANCASAGA-3′	[30] d
AD2	5′-AGWGNAGWANCAWAGG-3′	
AD3	5′-STTGNTASTNCTNTGC-3′	
AD4	5′-NTCGASTWTSGWGTT -3′	
AD5	5′-NGTCGASWGANAWGAA -3′	
AD6	5′-WGTGNAGWANCANAGA -3′	
AD7	5′-WTCTGNCTWANTANCT-3′	
LacA GSP1	5′-GGTAGCGATGCCGAATGAGGG-3′	This study^e^
LacA GSP2	5′- AGATGGGGCACTGGTTCACGA -3′	
SFP1	5′-CACGACACGCTACTCAACACACCA-3′	[31] f
SFP2	5′-AACACACCACCTCGCACAGC-3′	
SiteFinder1	5′-CACGACACGCTACTCAACACACCACCTCGCACAGCGTCCAAGCGGCCGCNNNNNNGCCT-3′	
SiteFinder2	5′-CACGACACGCTACTCAACACACCACCTCGCACAGCGTCCAAGCGGCCGCNNNNNNGCGC-3′	
LacA-RT-1	5′- ATGGCATTCCGTTCAGGC -3′	This study^g^
LacA-RT-2	5′- TTACTTGTCACCATCAGCAAGA -3′	
	R = A/G, Y = C/T, M = A/C, K = G/T, S = G/C, W = A/T, H = A/T/C, D = G/A/T, B = G/T/C, V = G/A/C, N = A/T/G/C.	

a Universal primers for amplification of 18S rDNA.

b Degenerate primers for amplification of the laccase sequence spanning the first and the fourth copper-binding motifs.

c Gene-specific primers for TAIL-PCR to amplify the 5′- and 3′-flanking sequences of the laccase fragment obtained with primers Cu I and Cu IV.

d Arbitrary degenerate primers for TAIL-PCR.

e Gene-specific primers for SiteFinding PCR to amplify the *LacA* promoter sequence.

f SiteFinders and their primers (SFP1 and SFP2) for SiteFinding PCR.

g Primers for amplification of the cDNA sequence of *LacA*.

### 2.3 Enzyme activity assay

Laccase activity was assayed according to a previously published method [23] with slight modifications. The reaction contained 25 mM citrate-phosphate buffer (pH 3.0) and 0.5 mM ABTS (Sigma-Aldrich, St. Louis, Missouri, USA), and increase in OD at 420 nm (ε = 36,000 M^−1 ^cm^−1^) was monitored for 5 min. One unit of enzyme activity (U) was defined as the amount of laccase required to oxidize 1 µmol ABTS per min. All assays were carried out in triplicate.

### 2.4 Protein purification

After cultivation in PDY medium for 4 d, the fermentation broth was harvested by paper filtration and then centrifuged (10,000 g, 20 min). The precipitate formed using 40 to 60% (NH_4_)_2_SO_4_ was collected by centrifugation (20,000 g, 20 min), resuspended in buffer A (25 mM Tris-HCl buffer, pH 7.5), and centrifuged (12,000 g, 5 min). The supernatant was desalted with a HiPrep 26/10 desalting column (GE Healthcare, Buckinghamshire, UK) and applied at 5 mL min^−1^ to a HiTrap DEAE column (5 mL) pre-equilibrated with buffer A. Adsorbed proteins were sequentially eluted with 0.1 M, 0.2 M and 1 M NaCl in buffer A. Fractions with laccase activity were pooled. (NH_4_)_2_SO_4_ was added until the final concentration was 1 M. The resulting sample was loaded at 1.0 mL min^−1^ onto a HiTrap Phenyl FF column (5 mL) pre-equilibrated with buffer A containing 1 M (NH_4_)_2_SO_4_. Adsorbed proteins were eluted with a linear 1.0–0 M (NH_4_)_2_SO_4_ gradient in buffer A. Fractions with laccase activity were collected, examined by SDS-PAGE and zymography and stored at 4°C. Deglycosylation of laccase by peptide *N*-glycosidase F (Takara, Dalian, China) was carried out. Protein identification with MALDI-TOF MS/MS was performed. Protein concentration was quantified by the Bradford method with bovine serum albumin as the standard [24].

### 2.5 SDS-PAGE analysis

SDS-PAGE [25] was conducted for molecular weight determination. For zymography analysis, nonreducing loading buffer (containing SDS but not DTT or β-mercaptoethanol) was employed, and the sample was not boiled before loading. After electrophoresis the gel was incubated in 10 mL sodium phosphate buffer (pH 6.0) containing 1% (v/v) Triton X-100 for 15–30 min for SDS removal [26] and then immersed in 10 mL fresh sodium phosphate buffer supplemented with 1 mM guaiacol for direct visualization of laccase activity on the gel.

### 2.6 Ultraviolet-visible (UV-Vis) absorption spectrum

The UV-Vis absorption spectrum of the purified laccase was recorded between 280 and 800 nm in buffer A.

### 2.7 Effects of pH and temperature on the activity and stability of purified LacA

The effect of pH was determined between pH 2.0 to 7.0 at 25°C. pH stability was studied by incubating the enzyme at pH 2.0–10.0 at 25°C for 105 h. Residual laccase activity was quantified with ABTS as substrate. 50 mM glycine-HCl buffer (pH 2.0), citrate-phosphate buffer (pH 2.5–6.5), sodium phosphate buffer (pH 7.0–8.0) and glycine-NaOH buffer (pH 9.0–10.0) were used.

For ascertaining optimum temperature, laccase activity was measured at optimum pH and temperatures from 25 to 80°C. Thermostability was analyzed by incubating the enzyme at different temperatures over a duration of 150 min, and residual activity was assayed with substrate ABTS at optimum pH and temperature. All experiments were performed in triplicate.

### 2.8 Effects of inhibitors and metal ions on the activity of purified LacA

Inhibitors including _L_-cysteine, DTT, EDTA, NaN_3_, SDS and kojic acid, and metal ions including Na^+^, K^+^, Li^+^, Cu^2+^, Ca^2+^, Mg^2+^, Mn^2+^, Zn^2+^, Fe^2+^ in form of sulfate, Cr^2+^ and Co^2+^ in form of nitrate, and Pb^2+^ in form of subacetate were examined. Individual inhibitor or metal ion was incorporated in the enzyme assay, and activity was determined at optimal temperature and pH. Enzyme activity in absence of inhibitors or metal ions was regarded as 100%.

### 2.9 Effects of various organic solvents on the activity and stability of LacA

5%, 10% or 25% of the individual solvents, namely methanol, ethanol, acetonitrile, dimethyl sulfoxide (DMSO) and *N*,*N*-dimethylformamide (DMF) was added to standard enzyme activity assay. For enzyme stability in presence of organic solvents, LacA was pre-incubated with 5%, 10% or 25% of each solvent at 25°C for 4 h, and residual enzyme activity was determined by using enzyme assay with ABTS as substrate.

### 2.10 Kinetic studies

Substrate specificity of LacA was determined in triplicate by using 1–1500 µM ABTS, and 25–2500 µM guaiacol, 2,6-dimethoxyphenol (2,6-DMP) and catechol at the respective optimum temperature and pH. Oxidation of the four substrates were measured for 5 min [27], [28]. The kinetic parameters were estimated based on nonlinear regression of the Michaelis-Menten equation using GraphPad Prism version 5.0 (GraphPad Software, San Diego, California, USA).

### 2.11 Decolorization

Dye decolorizing ability was evaluated using 25 dyes. The reaction mixture in 20 mL was composed of (in final concentration): 0.1 M citrate-phosphate buffer (pH 6.0), 0.2 or 2 U mL^−1^ laccase, 0.1 mM ACE or 12.5 mM kojic acid (if needed) and dyes. Decolorization was carried out in the dark at 28°C and 150 rpm for 24 h. The negative control had no enzyme. Decolorization was followed at the wavelength for each dye, determined from its absorbance spectrum in 0.1 M citrate-phosphate buffer between 280 and 800 nm. Decolorization (%) = [(Ai–At)/Ai]×100, where Ai is the initial absorption of the dye, and At is the absorption at the time of measurement.

The pH, conductivity and chemical oxygen demand of the textile effluent sample from Septwolves Group Co. in Fujian, China were 6.3, 537.2 µS cm^−1^ and 880.3 mg L^−1^, respectively. LacA was added to the sample at 20 U mL^−1^, and the mixture incubated at 28°C and 150 rpm for 6 d. Decolorization of the effluent was monitored every 24 h by measuring absorbance between 325 and 800 nm. All experiments were conducted in triplicate.

### 2.12 Cloning of the *LacA* sequences

All primers used are listed in Table 1. Mycelia were collected from 4-d-old *Cerrena* sp. HYB07 cultivated in PDY broth. Total RNA was extracted with TRIzol (Life Technologies, Grand Island, New York, USA). The RevertAid First Strand cDNA Synthesis Kit (Thermo Scientific, Waltham, Massachusetts, USA) was used to synthesize the first strands of cDNA. The degenerate primers Cu I and Cu IV (Table 1) designed according to the conserved amino acid sequences of the first and fourth copper-binding motifs in fungal laccases [29] were used to amplify laccase gene fragments from cDNA with high-fidelity DNA polymerase (Thermo Scientific, Waltham, Massachusetts, USA). PCR products were purified and inserted into pMD18-T vector. Recombinant vectors were transformed into *E. coli* TOP10 competent cells. Twenty clones were randomly selected and submitted to sequencing analysis (Life Technologies, Grand Island, New York, USA). All 20 clones corresponded to the same laccase gene, confirmed to be the gene encoding LacA by MALDI-TOF MS/MS analysis. The 5′- and 3′-flanking genetic sequences of the amplified laccase fragment were obtained by thermal asymmetric interlaced PCR (TAIL-PCR) [30] with HYB07 genomic DNA as the template, LacA SP1-6 as gene-specific primers and AD1-7 as arbitrary degenerate primers (Table 1) [30]. The full-length cDNA sequence was amplified with primers LacA-RT-1 and LacA-RT-2 (Table 1). The sequence has been submitted to GenBank with the accession number KF317949. SiteFinding PCR [31] was performed to further extend the promoter region of *LacA*, in which the gene-specific primers were LacA GSP1 and GSP2 (Table 1).

### 2.13 Bioinformatic analysis

The *LacA* sequences were analyzed using the Vector NTI program (Life Technologies, Grand Island, New York, USA) and BLAST (http://blast.ncbi.nlm.nih.gov/Blast.cgi) [32]. Signal peptide was predicted with SignalP 3.0 (http://www.cbs.dtu.dk/services/SignalP/) [33]. The theoretical isoelectric point (pI) and molecular weight (MW) were predicted by the Compute pI/Mw tool (http://web.expasy.org/compute_pi/) [34]. Potential *N*-glycosylation sites (Asn-X-Ser/Thr) were identified with ScanProsite (http://prosite.expasy.org/scanprosite/) [35]. Alignments of laccase proteins were generated with Clustal Omega (http://www.ebi.ac.uk/Tools/msa/clustalo/) [36]. The identities between selected fungal laccases were calculated in MEGA version 5.0 (http://www.megasoftware.net/) [37].

## Results

### 3.1 Species identification

**Figure 1:**
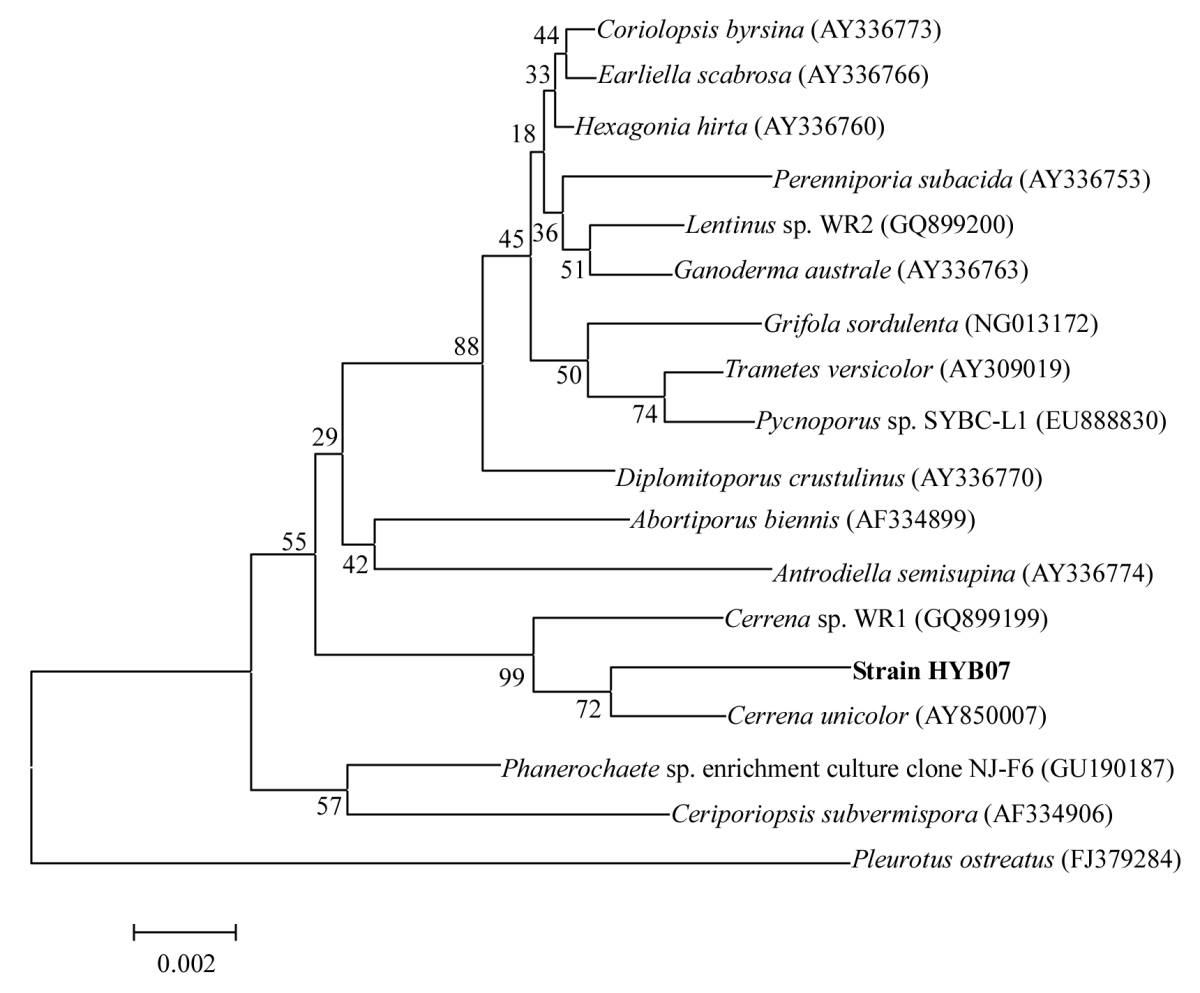
Phylogenetic relationships of *Cerrena* sp. HYB07 and related species based on 18S rDNA sequences. The numbers in parentheses are accession numbers of 18S rDNA sequences. Bootstrap values at nodes are percentages of 1,000 replicates. Scale bar indicates base substitutions/100 bases.

A fragment of the 18S rDNA gene of the target fungal strain HYB07 was amplified by PCR and sequenced, and HYB07 was classified based on its 18S rDNA sequence. A phylogeny tree (Fig. 1) was constructed, indicating HYB07 was closest to *Cerrena unicolor* (GenBank accession No. AY850007), sharing 99% sequence identity. The strain was named *Cerrena* sp. HYB07.

### 3.2 Production and purification of LacA

**Figure 2:**
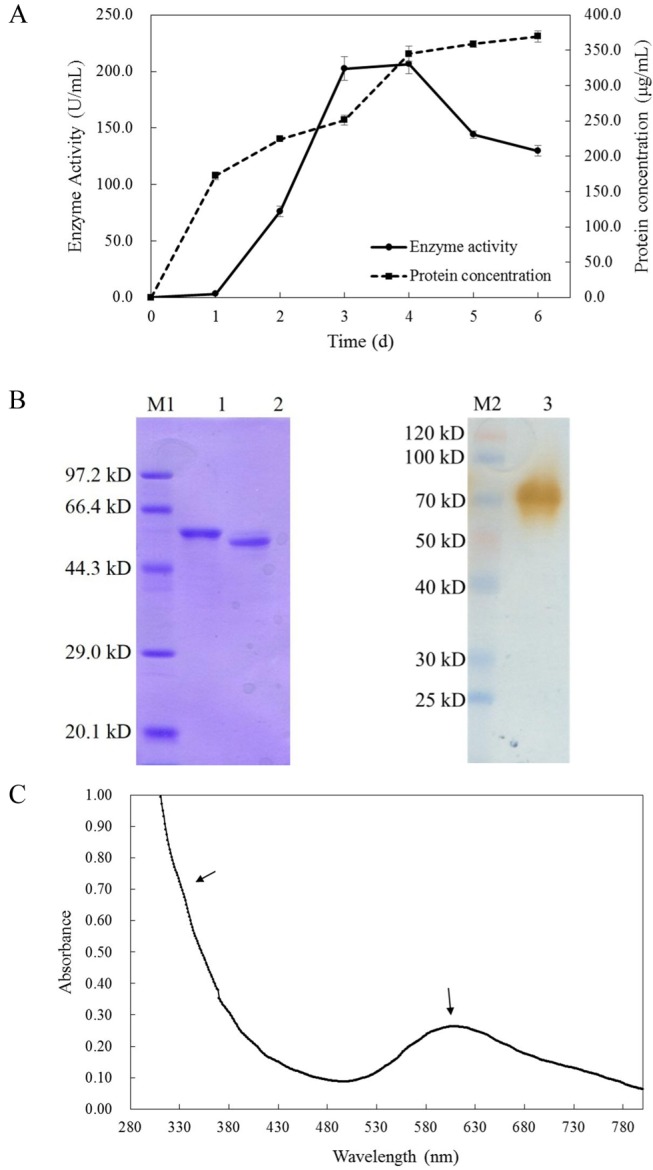
Purification and characterization of LacA from *Cerrena* sp. HYB07. (A) Time course of laccase activity and secreted protein levels produced by *Cerrena* sp. HYB07. Error bars represent standard deviations of triplicate experiments. (B) SDS-PAGE analysis of LacA. Lane M1, Protein molecular weight marker (Low) by Takara; lane 1, purified LacA; lane 2, purified LacA after peptide *N*-glycosidase F treatment; lane M2, Blue Plus II Protein Marker (Transgen, China); lane 3, purified LacA. Lanes 1 and 2 were stained with Coomassie Brilliant Blue R-250 after reducing electrophoresis; lane 3 was stained with guaiacol after non-reducing electrophoresis, and the sample was not heated before loading. (C) UV-Vis absorbance spectrum of purified LacA.

Laccase production by *Cerrena* sp. HYB07 in PDY liquid medium was monitored for 6 d in the shaking flask. With Cu^2+^ as inducer, maximum laccase activity (210.8 U mL^−1^) was reached on day 4. Specific activity (806.3 U mg^−1^) peaked on day 3 (Fig. 2A).

After (NH_4_)_2_SO_4_ precipitation, anion exchange and hydrophobic interaction chromatography, a monomeric laccase, designated as LacA, was purified 3.1-fold with 39.8% yield from the fermentation broth. The specific activity was 1952.4 U mg^−1^ (Table 2). Deglycosylation with peptide *N*-glycosidase F indicated *N*-glycosylation. The molecular weight before and after deglycosylation was 58.6 and 54.5 kDa, respectively, thus its glycosylation level was 7.2%. The molecular weight of ∼70 kDa on zymograph exceeded 58.6 kDa in SDS-PAGE (Fig. 2B).

**Table 2:** Purification of LacA from the culture supernatant of *Cerrena* sp. HYB07.

Purification step	Totalprotein(mg)	Totalactivity(U)	Specificactivity(U mg^−1^)	Purificationfold	Yield(%)
Culture supernatant	283.2	178,361.3	629.8	1.0	100
40–60% (NH_4_)_2_SO_4_precipitation	113.5	131,095.6	1155.0	1.8	73.5
DEAE FF	45.8	80,619.3	1760.2	2.8	45.2
Phenyl FF (High Sub)	36.4	71067.6	1952.4	3.1	39.8

The laccase solution was blue. UV-Vis absorption spectrum (Fig. 2C) showed a peak at 610 nm and a shoulder at 330 nm. The A280:A610 ratio was 20.6.

### 3.3 Effects of temperature and pH

**Figure 3:**
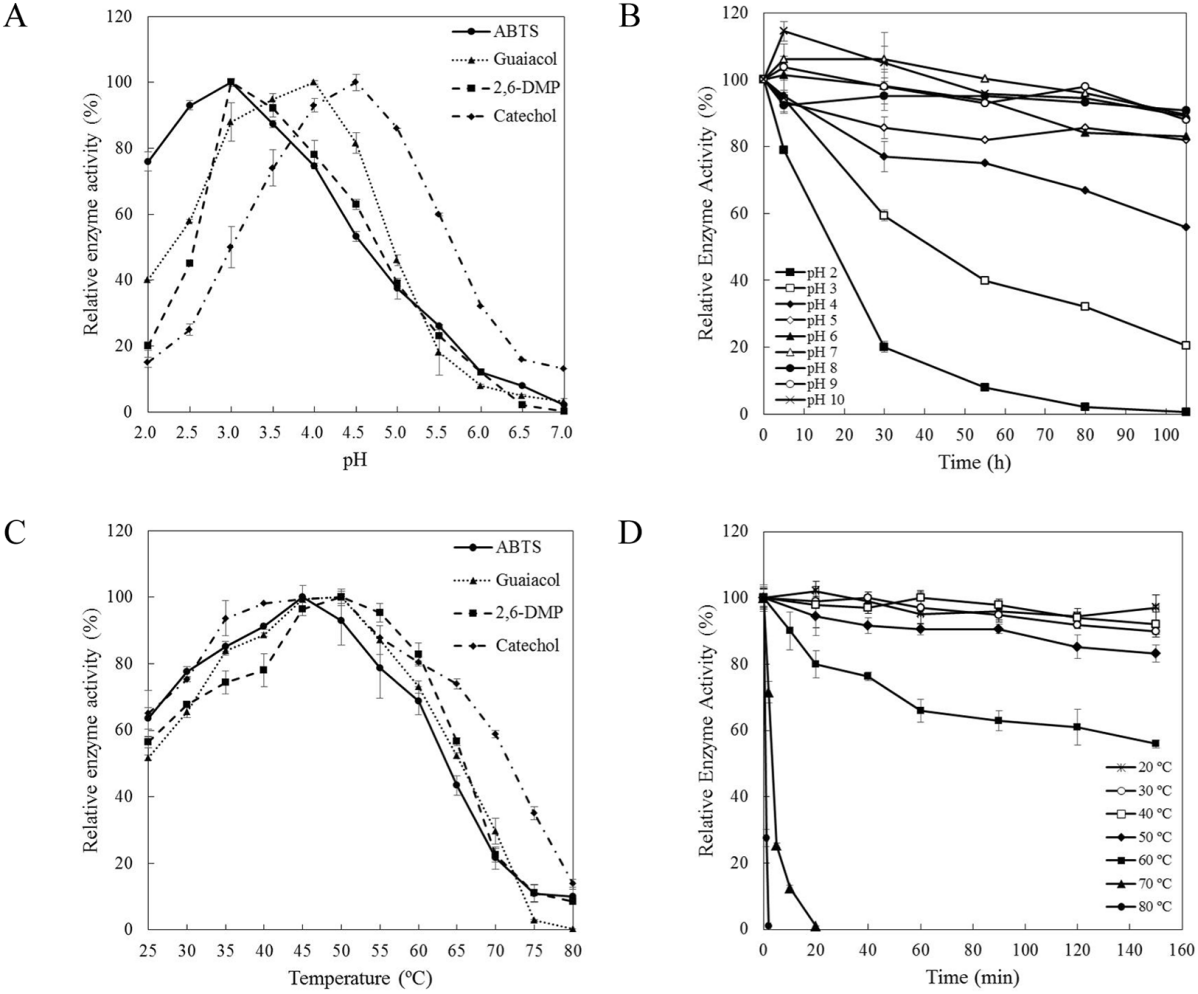
Effects of pH and temperature on activity and stability of LacA. (A) Effect of pH on LacA activity with ABTS, guaiacol, 2,6-DMP and catechol as substrates. (B) Effect of pH on LacA stability with ABTS as substrate. (C) Effect of temperature on LacA activity with ABTS, guaiacol, 2,6-DMP and catechol as substrate. (D) Effect of temperature on LacA stability with ABTS as substrate. Bars indicate standard deviations of triplicate determinations.

The pH optimum was 3.0 for ABTS and 2,6-DMP, 4.0 for guaiacol and 4.5 for catechol (Fig. 3A). After storage at pH 4.0 for 105 h, 56% residual enzyme activity was retained. At pH 5.0 or higher, >80% activity remained. In contrast, LacA activity decreased to only approximately 1% and 20% at the end of 105-h incubation at pH 2.0 and 3.0, with *t*_1/2_ being 18 h and 43 h, respectively (Fig. 3B).

Optimal temperature was 45°C with ABTS, 50°C with 2,6-DMP and 45–50°C with guaiacol and catechol. The enzyme displayed >50% of maximal activity between 25 and 65°C against the four substrates except that at 65°C for ABTS, the relative activity of LacA was only 45% (Fig. 3C). After incubation for 150 min at 50 and 60°C, approximately 85% and 56% of the original enzyme activity was retained, respectively. LacA was inactivated after 20 min at 70°C and 2 min at 80°C (Fig. 3D).

### 3.4 Effects of inhibitors and metal ions

Relative laccase activity was dramatically reduced in the presence of _L_-cysteine (to 5.5%), DTT (to 5.9%), sodium azide (to 9.2%) and SDS (to 11.4%) at the concentration of 0.1 mM. The laccase-specific inhibitor kojic acid at 12.5 mM reduced LacA activity by 50%. No significant activity loss was observed with 10 mM EDTA (Table 3).

**Table 3:** Effect of inhibitors and metal ions on LacA activity.

Inhibitor	Concentration (mM)	Relative activity (%)	Metal ion	Concentration (mM)	Relative activity (%)
_L_-Cysteine	0.01	87.5±1.7	Na^+^	10	115.1±0.9
	0.1	5.5±0.1	K^+^	10	109.8±1.7
DTT	0.01	38.4±7.1	Li^+^	10	48.8±0.9
	0.1	5.9±0.7	Cu^2+^	10	101.9±4.3
EDTA	1	95.4±3.2	Ca^2+^	10	113.6±2.0
	10	82.4±2.4	Mg^2+^	10	111.6±3.2
NaN_3_	0.01	30.2±2.5	Mn^2+^	10	117.3±2.3
	0.1	9.2±1.5	Zn^2+^	10	111.0±3.9
SDS	0.01	80.8±0.9	Fe^2+^	10	3.8±0.8
	0.1	11.4±1.2	Cr^2+^	10	99.0±1.1
Kojic acid	12.5	49.3±1.8	Co^2+^	10	99.8±4.1
	50	31.7±3.2	Pb^2+^	10	93.8±3.7

Values represent mean ± standard deviation (*n* = 3).

At 10 mM, Fe^2+^ exerted the strongest inhibition, followed by Li^+^, while Na^+^, K^+^, Ca^2+^, Mg^2+^, Mn^2+^ and Zn^2+^ were slightly stimulatory. Cu^2+^, Cr^2+^, Co^2+^ and Pb^2+^ had no significant effect (Table 3).

### 3.5 Effects of various organic solvents

When individual water-miscible organic solvent was added at 5 and 10% final concentration, 102.5–72.7% activity was retained. Activity was compromised in all solvents at 25%; >70% activity remained with DMF and ethanol, approximately 60% activity remained with other three organic solvents (Table 4).

**Table 4:** Effect of organic solvents on the activity and stability of purified LacA.

Organic solvent	Concentration (%)	Relative Activity^a^ (%)	Residual Activity^b^ (%)
Methanol	5	92.9±5.3	92.7±1.1
	10	72.7±6.7	107.7±3.1
	25	57.3±6.9	116.5±5.9
Ethanol	5	101.0±4.7	94.0±7.2
	10	83.1±2.9	101.0±4.5
	25	70.9±3.5	100.2±3.0
Acetonitrile	5	102.5±4.2	105.0±2.8
	10	96.8±1.9	95.9±5.1
	25	66.2±2.0	50.4±2.6
DMSO	5	91.1±2.1	98.1±4.7
	10	77.3±4.4	116.8±3.1
	25	58.7±1.8	124.9±2.4
DMF	5	95.1±3.7	95.9±2.1
	10	82.5±2.5	100.7±0.9
	25	78.9±2.8	94.4±1.3

Values represent mean ± standard deviation (*n* = 3).

a Enzyme activity was measured with individual organic solvent included in the enzyme activity assay mixture at the concentration indicated.

b Purified LacA was incubated with individual organic solvent at the concentration indicated for 4 hours at 25°C before the standard enzyme activity assay was conducted.

LacA was generally stable in the presence of the organic solvents. Residual activity was determined after incubation with each solvent at 5, 10 or 25% for 4 h at 25°C. An exception was exposure to 25% acetonitrile which was detrimental to activity. LacA was more stable in methanol and DMSO than in aqueous solution.

### 3.6 Kinetic properties of purified LacA

Initial rate kinetic analyses were performed with ABTS, guaiacol, 2,6-DMP and catechol at the respective optimal temperature and pH values (Table 5). The lowest *K*_m_ value was found for ABTS (93.4 µM), so were the highest turnover rate (*k*_cat_) and catalytic efficiency (*k*_cat_/*K*_m_) values (2468.0 s^−1^ and 26.4 µM^−1 ^s^−1^, respectively). Catalytic efficiency on ABTS was 7.5 times of that on 2,6-DMP, 22 times of that on catechol and 65 times higher than that on guaiacol. Catechol had the highest *K*m value (528.8 µM), followed by guaiacol (299.8 µM) and 2,6-DMP (200.4 µM).

**Table 5:** Substrate specificity of LacA.

Substrate	Wavelength (nm)	ε (M^−1 ^cm^−1^)	*K*_m_ (µM)	*k*_cat_ (s^−1^)	*k*_cat_/*K*_m_ (µM^−1 ^s^−1^)
ABTS	420	36,000	93.4	2468.0	26.4
Guaiacol	470	26,600	299.8	128.1	0.4
2,6-DMP	468	49,600	200.4	703.7	3.5
Catechol	400	1,260	528.8	626.0	1.2

### 3.7 Dye and real textile effluent decolorization by LacA

Decolorizing capability of purified LacA on industrial and laboratory dyes was evaluated at a low enzyme activity of 0.2 U mL^−1^ (Table 6). Without the help of small redox mediators, LacA was able to effectively decolorize dyes of different classes, some of which were notoriously recalcitrant to biodegradation, e.g., the indigo dye carmine, triphenylmethane dye malachite green, anthraquinone dye Remazol Brilliant Blue R (RBBR), and azo dyes acid violet 7 and orange II. Among the 25 dyes studies, 13 could be oxidized by 0.2 U mL^−1^ LacA with efficiencies higher than 50%. It was worth pointing out that although 24 h was arbitrarily selected for decolorization measurements, decolorization of some dyes proceeded faster than others. For example, decolorization of RBBR, indigo carmine and Evans blue was completed within 30 min, but took approximately 2 hours for malachite green and brilliant green (data not shown). A higher enzyme activity of 2.0 U mL^−1^ helped improve decotablorization efficiencies to different extents. For instance, the decolorization rate increased from 28.8% to 92.4% for Coomassie Brilliant Blue R-250, whereas for Congo red, only a modest improvement (from 33.0% to 57.2%) was seen. On the contrary, for four dyes, namely basic fuchsin, crystal violet, methylene blue and Rhodamine B, there were no significant changes. Therefore, LacA alone at 2.0 U mL^−1^ could decolorize 19 dyes with efficiencies higher than 50%. For the nine dyes with decolorization efficiencies lower than 60%, we further evaluated their removal by LacA in the presence of 0.1 mM ACE, a natural laccase mediator. Seven of the nine dyes were efficiently decolorized by the LacA-ACE system except for Rhodamine B and methylene blue. For dyes such as methyl orange and neutral red which could already be partially decolorized by LacA, their decolorization rates were enhanced by ACE (from 53.2% and 28.0% to 92.3% and 80.1%, respectively). In the cases of crystal violet and basic fuchsin, however, ACE was required for their decolorization, since neither was decolorized by LacA alone at 2.0 U mL^−1^. Even for dyes with over 80% decolorization efficiencies, inclusion of the laccase-specific inhibitor kojic acid in reactions decreased decolorization efficiencies to 4.2–28.4%, suggesting that LacA was responsible for dye decolorization.

**Table 6:** Decolorization of the dyes by LacA.

	Concentration(mg L^−1^)	λmax	Decolorization(%)		
			0.2 U mL^−1^	2 U mL^−1^	2 U mL^−1^+ACE
Acid Chrome Blue K	25	547	41.3±9.1	**93.5±2.8**	ND
Acid Fuchsin	50	545	10.3±1.2	25.5±6.6	**64.1±2.3**
Acid Violet 7	25	519	**87.8±2.1**	**96.4±0.9**	ND
Aniline Blue	100	585	**78.2±1.8**	**78.2±1.7**	ND
Basic Fuchsin	25	543	6.9±2.1	9.9±2.6	**81.4±0.7**
Brilliant Green	25	622	**90.6±4.3**	**91.7±4.7**	ND
Bromocresol Green	25	614	12.4±2.7	**58.3±2.5**	**96.8±0.3**
Bromocresol Purple	25	588	**78.5±2.4**	**89.7±1.6**	ND
Bromothymol Blue	200	427	**73.7±0.8**	**68.4±4.6**	ND
Congo Red	50	478	33.0±1.4	**57.2±4.5**	**80.6±1.9**
Coomassie BrilliantBlue R-250	25	559	28.8±5.8	**92.4±4.7**	ND
Crystal Violet	25	554	5.8±2.1	6.4±2.9	**87.0±1.7**
Eriochrome Black T	100	576	**84.2±1.5**	**85.3±1.7**	ND
Evans Blue	25	597	**89.3±1.6**	**94.0±0.6**	ND
Indigo Carmine	50	609	**95.3±2.1**	**97.7±0.4**	ND
Malachite Green	25	618	**94.1±7.4**	**96.3±1.2**	ND
Methylene Blue	25	597	4.1±1.9	6.7±0.7	5.6±2.2
Methyl Orange	50	463	18.6±2.9	**53.2±3.7**	**92.3±0.9**
Methyl Red	25	429	25.2±2.9	**62.8±0.8**	ND
Neutral Red	25	517	13.4±1.9	28.0±2.4	**80.1±3.8**
Orange II	25	483	**79.4±3.3**	**87.3±1.4**	ND
RBBR	100	594	**80.8±2.0**	**96.9±0.3**	ND
Rhodamine B	25	546	3.8±1.6	6.7±1.3	5.5±1.2
Sudan IV	200	536	**97.9±1.2**	**98.4±0.9**	ND
Victoria Blue B	25	612	**59.0±1.4**	**92.3±1.2**	ND

Values represent mean ± standard deviation (*n* = 3).

Decolorization efficiencies higher than 50% are highlighted in bold.

ND, not determined.

**Figure 4:**
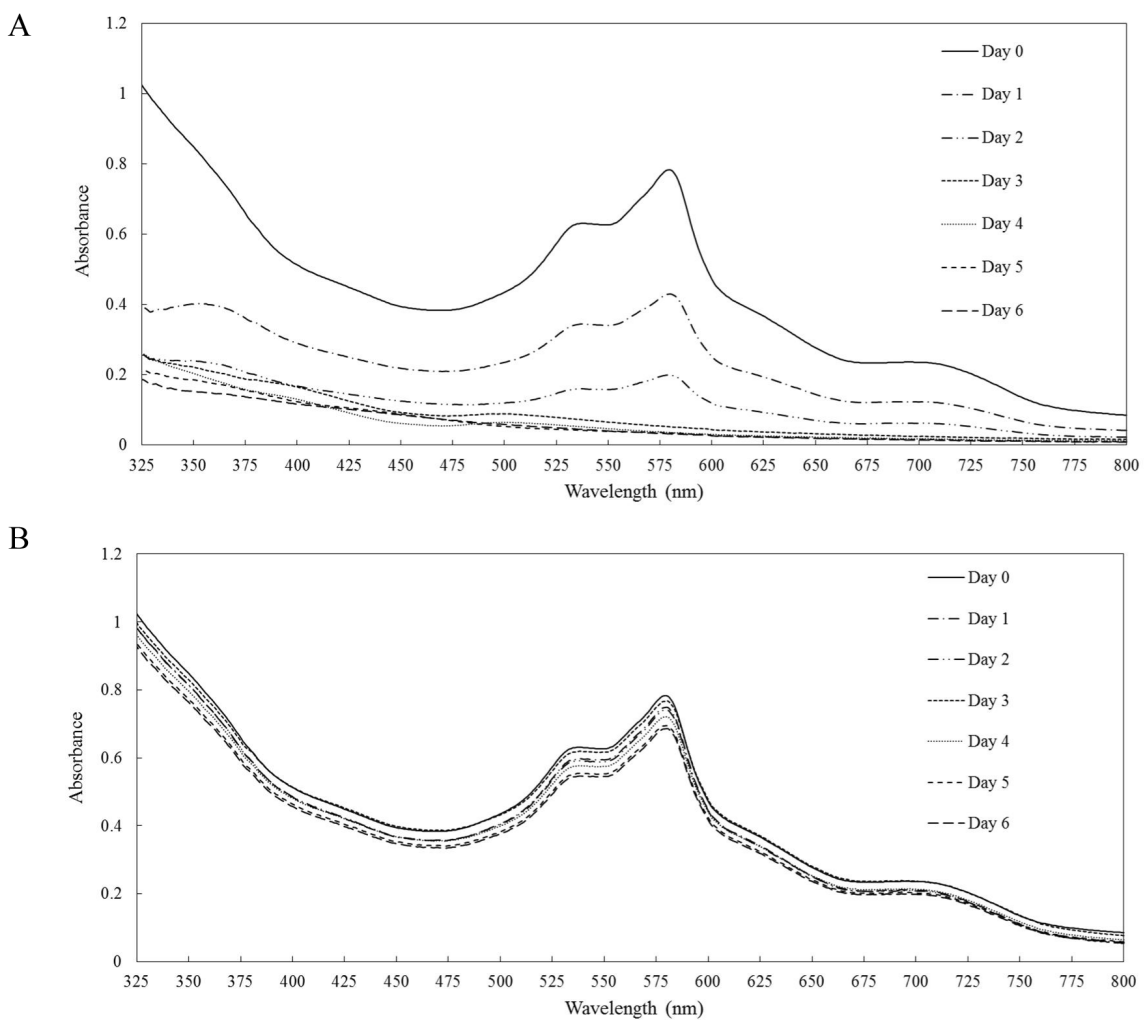
LacA-mediated decolorization of a real textile effluent. (A) Absorption spectra of the real textile effluent during LacA-mediated decolorization recorded every 24 h for 6 d. (B) Absorption spectra of the real textile effluent without LacA.

Decolorization of the real textile effluent by LacA predominantly occurred within the first 3 d, accompanied by flattening of the absorption spectrum (Fig. 4A). A negative control was conducted in parallel, and there was little decrease in the absorbance in the absence of LacA (Fig. 4B).

### 3.8 *LacA* gene cloning and analysis

A fragment of *LacA* between its first and fourth copper-binding motifs was amplified from *Cerrena* sp. HYB07 cDNA. TAIL-PCR was adopted to clone the 5′ and 3′-flanking genetic sequences of the fragment, based on which the full-length gene sequence of *LacA* was obtained. The 3,685-bp DNA sequence consisted of an 892 bp 5′-uncoding region, a 2168-bp gene sequence and a 625-bp 3′-uncoding region. The full-length cDNA sequence (1,551 bp) was subsequently obtained. The *LacA* cDNA corresponded to purified LacA, as confirmed by MALDI-TOF MS/MS analysis (Fig. S1). The coding region was interrupted by 11 introns between 50–65 bp, and all splicing junctions adhered to the GT-AG rule.

LacA consisted of 516 amino acids, with the first 21 residues being the signal peptide, and 3 putative *N*-glycosylation sites at positions 453, 489 and 495. The molecular weight of mature LacA was predicted to be 52.9 kDa, close to the observed 54.5 kDa. Calculated pI was 5.6.

**Figure 5:**
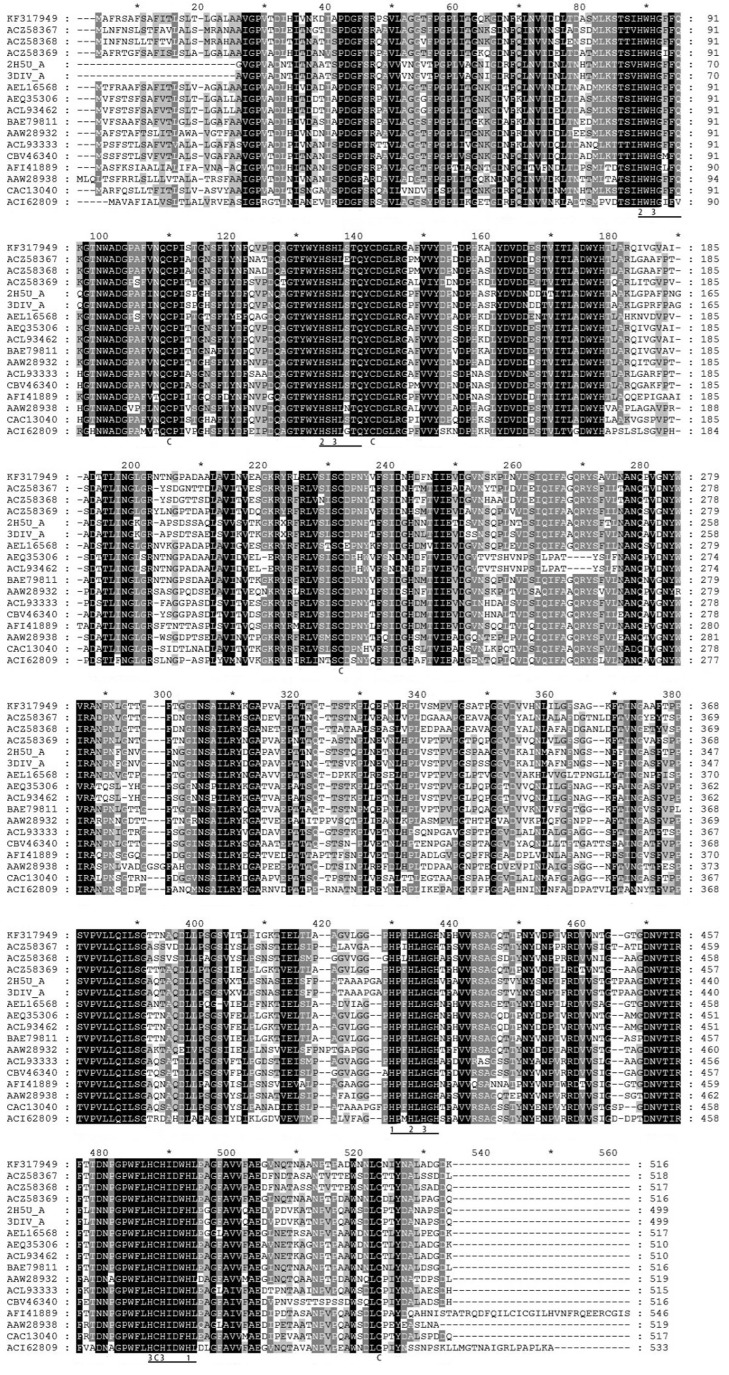
Alignment of deduced amino acid sequence of LacA with other laccases (indicated by the GenBank accession numbers). Laccases used in alignment are: *Cerrena* sp. HYB07 LacA: KF317949; *Cerrena* sp. WR1 Lcc1: ACZ58367; *Cerrena* sp. WR1 Lcc2: ACZ58368; *Cerrena* sp. WR1 Lcc3: ACZ58369; *Cerrena maxima* laccase chain A: 2H5U_A; *Cerrena maxima* laccase chain A: 3DIV_A; *Cerrena* sp. CTL-2011 laccase: AEL16568; *Cerrena unicolor* laccase: AEQ35306; *Cerrena unicolor* Lac1: ACL93462; *Spongipellis* sp. FERM P-18171 laccase 1 precursor: BAE79811; *Panus rudis* laccase A: AAW28932; *Rigidoporus microporus* laccase: ACL93333; *Meripilus giganteus* laccase: CBV46340; *Steccherinum murashkinskyi* laccase 2: AFI41889; *Trametes* sp. 420 laccase C: AAW28938; *Coriolopsis trogii* laccase: CAC13040; *Pleurotus eryngii* laccase: ACI62809. Four conserved copper binding domains are underlined. Conserved His residues are numbered, and conserved Cys residues are labeled.

The deduced amino acid sequence of LacA was aligned with other fungal laccases, including 8 *Cerrena* laccases (Fig. 5). The LacA protein possessed four conserved copper-binding motifs typical of fungal laccases, Cu I (HWHGFFQ), Cu II (HSHLSTQ), Cu III (HPFHLHGH) and Cu IV (HCHIDWHL), as well as 10 conserved His involved in copper atom coordination and 5 conserved Cys residues. LacA was most similar to and exhibited 86% identity to the laccase 1 precursor from *Spongipellis* sp. FERM P-18171 (BAE79811), followed by Lac1 from *Cerrena unicolor* (ACL93462) with 81% identity, Lcc3 from *Cerrena* sp. WR1 (ACZ58369) with 78% identity and laccase from *Cerrena* sp. CTL-2011 (AEL16568) with 74% identity. On the other hand, LacA was more distantly related to *Trametes* and *Pleurotus* laccases (with no higher than 70% identity).

**Figure 6:**
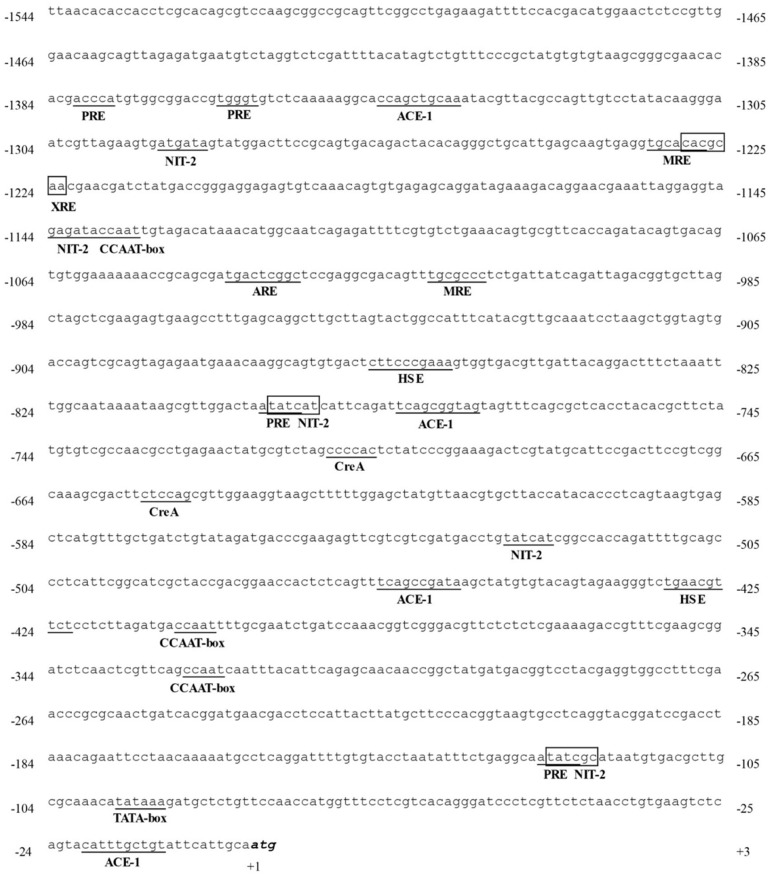
Promoter analysis of *LacA* gene. First nucleotide of the start codon ATG is designated as +1. Putative *cis*-acting responsive elements are underlined/boxed and labeled according to following abbreviations. ACE1: ACE1 copper-responsive element; ARE: antioxidant response element; CreA: CreA-binding site; HSE: heat shock response element; MRE: metal response element; NIT2: consensus sequences for binding of NIT2 transcription factor; PRE: putative response element; XRE: xenobiotic response element. Putative CCAAT and TATA boxes are underlined.

SiteFinding PCR was adopted to further extend the 5′-flanking region of *LacA*, rendering a region of 1,544 bp upstream of the start codon, referred to as the *LacA* promoter. Bioinformatics analysis revealed multiple putative *cis*-acting transcription regulation sites within the *LacA* promoter sequence in both orientations (Fig. 6). A TATA box was located 92 bp upstream from the start codon ATG, and three CCAAT boxes were found at positions −324, −405 and −1134. The *LacA* promoter contained two metal response elements (MREs) with the consensus sequence TGCRCNC at positions −1013 and −1227 and one xenobiotic response element (XRE) with the core sequence TNGCGTG [38] at −1223. Apart from MREs and XREs, *LacA* had many ACE1 copper-responsive transcription factor binding sites, consisting of the HWHNNGCTGD or NTNNHGCTGN core [39], at positions −11, −456, −774 and −1336, respectively. In addition, one antioxidant response element (ARE) adhering to the consensus sequence TGACNNNGC [40] was present at −1035 in the *LacA* promoter. Putative response elements (PREs) [38], often found in basidiomycete laccase promoter sequences, were also present within the 5′-flanking sequence of *LacA*: a TGGGT was located at position −1360, an inverted one at −1377, and two ATATC at −122 and −795. Furthermore, two heat shock response elements (HSEs) composed of alternately oriented NGAAN repeats [41] were found at −422 and −857 in the *LacA* promoter. *LacA* also contained multiple transcription factor binding sites involved in nitrogen and carbon regulation. Two putative CreA-binding sites (SYGGRG) were found at −648 and −706, and five NIT2 binding sites adhering to sequence TATCDH [5] were scattered at −120, −525, −793, −1139 and −1286. No stress response elements (STREs) with the consensus sequence of CCCCT or Sp-1 transcription factor recognition site (GGGCGG) [39] were identified in the *LacA* promoter sequence.

## Discussion

Although laccases have industrial and environmental health implications, commercial applications are limited by low enzyme yields, high costs and tolerance to extreme conditions. Herein, a new *Cerrena* sp. strain HYB07 produced over 200 U mL^−1^ laccase activity after cultivation for only 3 d with Cu^2+^ as the inducer. The high laccase yield and short production period of HYB07 would be advantageous for application and commercialization, since many fungal laccase producers require longer production periods [14]. For *C. unicolor* VKMF-3196, 15 U mL^−1^ was obtained on the 8^th^ day of cultivation [20]. Enhanced laccase production by *Cerrena unicolor* strain MTCC 5159 was observed with inducers, effluents and synthetic dyes, and a textile effluent was the most effective inducer, resulting in 85.8 U mL^−1^ after 12 d [18]. For *Cerrena* sp. WR1, maximal production was 202 U mL^−1^ after 13-d cultivation under Cu^2+^ and 2,5-xylidine induction [9]. For *Cerrena unicolor* C-139 [15], [16], a production of 250.0 U mL^−1^ was reached after submerged culture for 7 d, and activity culminated at 450 U mL^−1^ after 14 d. We aim at optimizing the fermentation medium and conditions of HYB07, and even higher laccase yields are anticipated.

Apart from easy production, the novel laccase LacA purified from the fermentation broth of HYB07 showed low substrate specificity and strong decolorizing ability. LacA is a blue multicopper oxidase; the absorption peak at 610 nm and shoulder around 330 nm indicated the presence of type I (responsible for the blue color) and type III copper ions. The A280/A610 ratio of 20.6 is similar to other laccases [42].

LacA manifested 60–81% identity to other *Cerrena* laccases. Comparison with reported fungal laccases enabled speculation on five conserved Cys residues: (1) Cys-106 and Cys-505 formed a disulfide; (2) Cys-138 and Cys-226 formed another disulfide; and (3) Cys-470 was a ligand to type I copper domain [43]. Phe located ten residues downstream of Cys-470, indicating LacA is a Class 3 laccase likely with a high redox potential (E^0^) [44], [45]. This inference is reinforced by “Leu-Glu-Ala” located at positions +6 to +8 of Cys-470, also adjacent to His-475. Site-directed mutagenesis on fungal laccases disclosed “Leu-Glu-Ala” and “Val-Ser-Gly” ensue in high and low redox potential, respectively [43], [45].

Like other laccase promoters [5], [39], [41], [46], putative regulatory elements found in the promoter sequence suggest regulation of *LacA* transcription by metal ions, aromatic compounds, phenolic antioxidants, etc. Nitrogen and carbon may play a role in *LacA* expression, as inferred from CreA and NIT2 binding sites in the promoter region. *LacA* expression may be repressed by glucose since CreA is a major regulator in carbon catabolite repression and NIT2 activates gene expression when nitrogen is limiting [39], [41]. *LacA* promoter is reminiscent of reported laccases whose transcription is regulated by metal ions, aromatic compounds related to lignin or lignin derivatives and nutrient nitrogen and carbon [18], [39], [41] and forms the basis for elucidating the functional correlation between possible regulatory elements and *LacA* expression.

LacA had a broad substrate range, as manifested by the kinetic and decolorization studies. Among the four compounds used for kinetic experiments, ABTS was the preferred substrate for LacA, and LacA had the lowest affinity for catechol. The specific activity of LacA against ABTS was 1.9–13.9 times as high as the specific activity of laccases from *Cerrena unicolor* (CFC-120) [17], *Cerrena unicolor* strain 137 [14] and *Cerrena* sp. WR1 [9]. In addition, compared with an alkali-resistant and metal-tolerant laccase Tplac from *Trametes pubescens* [11], the specific activity of LacA was over 100-fold higher, along with a higher affinity and catalytic efficiency towards ABTS. Despite the common use of ABTS in laccase assays, LacA also reacted with guaiacol and 2,6-DMP, two lignin building blocks. Though the catalytic efficiency of LacA on ABTS was only 1/10 of Lcc3 from *Cerrena* sp. WR1 (which has a small *K*_m_ value), its catalytic efficiencies on guaiacol, 2,6-DMP and catechol were approximately 2.4, 12.5 and 9.2-fold of the corresponding efficiencies of Lcc3 [9]. Furthermore, the catalytic efficiency of LacA on 2,6-DMP was also higher than those of both laccases from *Cerrena unicolor* strain 137 [14]. The speculated high redox potential and low substrate specificity of LacA can be beneficial for its practical application, which was corroborated by LacA-catalyzed breakdown of structurally different dyes. Since dyes in the same class may not respond equally to the same laccase [47], it may be necessary to test the decolorizing efficiencies of individual dyes of interest with specific laccases. This was also true for LacA. LacA alone decolorized malachite green with >90% efficiency, whereas LacA degraded crystal violet only in presence of a laccase mediator. Besides degrading a variety of dyestuffs, LacA also decolorized a real textile effluent. Hence, LacA holds great promise for applications in biodegradation and bioremediation, especially treatment of dye effluents.

The activity of LacA over 25–65°C helps reducing application costs. LacA displayed strong decolorizing ability at 28°C. LacA was more thermostable than alkali-resistant and metal-tolerant *Trametes pubescens* Tplac [11] whose unusual instability at 10/20°C causes handling and storage problems. LacA was also more robust at 60°C than *Cerrena* sp. WR1 Lcc3 [9], *Shiraia* sp. SUPER-H168 laccase [10] and *Streptomyces sviceus* Ssl1 [27], whose *t*_1/2_ at 60°C are 40, 120 and 88 min, respectively. LacA was more stable at pH 4.0 than *Cerrena* sp. WR1 Lcc3 [9] and *Trametes pubescens* Tplac [11] and at various pH values than laccases from *Cerrena unicolor* C-139[16] and MTCC5159 [19]. Furthermore, LacA was highly tolerant of organic solvents, a quality valuable for industrial processes such as organic synthesis. The high production yield, robustness, broad substrate specificity, and remarkable dye decolorizing capacity make LacA and fermentation broth of *Cerrena* sp. HYB07 an attractive and affordable candidate for applications in a diversity of industries including biodegradation and bioremediation, textile, paper and pulp.

## Conclusion

A new *Cerrena* sp. strain HYB07 was reported, which produced over 200 U mL^−1^ laccase activity after 3-d cultivation in the shaking flask. The high laccase yield and short production period of HYB07 are advantageous for application and commercialization. A major laccase, namely LacA, was purified from the fermentation broth of HYB07 and showed sequence homology to other *Cerrena* laccases. The promoter sequence of *LacA* contained many putative regulatory elements. Biochemical characterization revealed that LacA had high specific activity, low substrate specificity, strong decolorizing ability, thermo- and pH-stability, and tolerance of organic solvents. Further work to explore LacA’s application potentials in various industrial processes, such as biodegradation, dye effluent decolorization and detoxification, textile finishing and organic synthesis, is warranted.

## Supporting Information

Figure S1 **The result of the MALDI-TOF MS/MS analysis of the purified LacA protein.**
(DOCX)

## References

[1] Chung K-T, Stevens SE Jr (1993) Degradation azo dyes by environmental microorganisms and helminths. Environ Toxicol Chem 12: 2121–2132.

[2] Wong Y, Yu J (1999) Laccase-catalyzed decolorization of synthetic dyes. Water Res 33: 3512–3520.

[3] Couto SR, Herrera JLT (2006) Industrial and biotechnological applications of laccases: A review. Biotechnol Adv 24: 500–513. 16716556 10.1016/j.biotechadv.2006.04.003

[4] Murugesan K, In-Hee Y, Young-Mo K, Jong-Rok J, Yoon-Seok C (2009) Enhanced transformation of malachite green by laccase of *Ganoderma lucidum* in the presence of natural phenolic compounds. Appl Microbiol Biotechnol 82: 341–350. 19130052 10.1007/s00253-008-1819-1

[5] Zhuo R, Ma L, Fan F, Gong Y, Wan X, et al (2011) Decolorization of different dyes by a newly isolated white-rot fungi strain *Ganoderma* sp.En3 and cloning and functional analysis of its laccase gene. J Hazard Mater 192: 855–873. 21733624 10.1016/j.jhazmat.2011.05.106

[6] Abadulla E, Tzanov T, Costa S, Robra K-H, Cavaco-Paulo A, et al (2000) Decolorization and detoxification of textile dyes with a laccase from *Trametes hirsuta* . Appl Environ Microbiol 66: 3357–3362. 10919791 10.1128/aem.66.8.3357-3362.2000 PMC92155

[7] Arora DS, Sharma RK (2010) Ligninolytic fungal laccases and their biotechnological applications. Appl Biochem Biotechnol 160: 1760–1788. 19513857 10.1007/s12010-009-8676-y

[8] Baldrian P (2006) Laccases-occurrence and properties. FEMS Microbiol Rev 30: 215–242. 16472305 10.1111/j.1574-4976.2005.00010.x

[9] Chen S-C, Wu P-H, Su Y-C, Wen T-N, Wei Y-S, et al (2012) Biochemical characterization of a novel laccase from the basidiomycete fungus *Cerrena* sp. WR1. Protein Eng Des Sel 25: 761–769. 23081836 10.1093/protein/gzs082

[10] Yang Y, Ding Y, Liao X, Cai Y (2013) Purification and characterization of a new laccase from *Shiraia* sp.SUPER-H168. Process Biochem 48: 351–357.

[11] Si J, Peng F, Cui B (2013) Purification, biochemical characterization and dye decolorization capacity of an alkali-resistant and metal-tolerant laccase from *Trametes pubescens* . Bioresour Technol 128: 49–57. 23196221 10.1016/j.biortech.2012.10.085

[12] Piontek K, Antorini M, Choinowski T (2002) Crystal structure of a laccase from the fungus *Trametes versicolor* at 1.90-Å resolution containing a full complement of coppers. J Biol Chem 277: 37663–37669. 12163489 10.1074/jbc.M204571200

[13] Pezzella C, Autore F, Giardina P, Piscitelli A, Sannia G, et al (2009) The *Pleurotus ostreatus* laccase multi-gene family: isolation and heterologous expression of new family members. Curr Genet 55: 45–57. 19034452 10.1007/s00294-008-0221-y

[14] Michniewicz A, Ullrich R, Ledakowicz S, Hofrichter M (2006) The white-rot fungus *Cerrena unicolor* strain 137 produces two laccase isoforms with different physico-chemical and catalytic properties. Appl Microbiol Biotechnol 69: 682–688. 15983808 10.1007/s00253-005-0015-9

[15] Janusz G, Rogalski J, Szczodrak J (2007) Increased production of laccase by *Cerrena unicolor* in submerged liquid cultures. World J Microbiol Biotechnol 23: 1459–1464.

[16] Songulashvili G, Jimenéz-Tobón GA, Jaspers C, Penninckx MJ (2012) Immobilized laccase of *Cerrena unicolor* for elimination of endocrine disruptor micropollutants. Fungal Biology 116: 883–889. 22862916 10.1016/j.funbio.2012.05.005

[17] Kim Y, Cho N-S, Eom T-J, Shin W (2002) Purification and characterization of a laccase from *Cerrena unicolor* and its reactivity in lignin degradation. Bull Korean Chem Soc 23: 985–989.

[18] D'Souza DT, Tiwari R, Sah AK, Raghukumar C (2006) Enhanced production of laccase by a marine fungus during treatment of colored effluents and synthetic dyes. Enzyme Microb Technol 38: 504–511.

[19] D’Souza-Ticlo D, Sharma D, Raghukumar C (2009) A thermostable metal-tolerant laccase with bioremediation potential from a marine-derived fungus. Mar Biotechnol 11: 725–737. 19283431 10.1007/s10126-009-9187-0

[20] Lisova ZA, Lisov AV, Leontievsky AA (2010) Two laccase isoforms of the basidiomycete *Cerrena unicolor* VKMF-3196. Induction, isolation and properties. J Basic Microbiol 50: 72–82. 20175123 10.1002/jobm.200900382

[21] Lin J, Lan S, Yang J, Li R, Ye X (2013) Screening of fungal strains with high yield of laccase and studying on the laccase-producing conditions. J Chin Inst Food Sci Technol 13: 110–116.

[22] White TJ, Bruns T, Lee S, Taylor J (1990) Amplification and direct sequencing of fungal ribosomal RNA genes for phylogenetics. In: Innis MA, Gelfand DH, Sninsky JJ, White TJ, editors. PCR protocols: A guide to methods and applications. New York, N. Y.: Academic Press. 315–322.

[23] Kwang-Soo S, Chang-Jin K (1998) Properties of laccase purified from nitrogen limited culture of white-rot fungus *Coriolus hirsutus* . Biotechnol Tech 12: 101–104.

[24] Bradford MM (1976) A rapid and sensitive method for the quantitation of microgram quantities of protein utilizing the principle of protein-dye binding. Anal Biochem 72: 248–254. 942051 10.1016/0003-2697(76)90527-3

[25] Sambrook J, Russell D (2001) Molecular cloning: A laboratory manual. Cold Spring Harbor, New York: Cold Spring Harbor Laboratory Press.

[26] Bischoff KM, Shi L, Kennelly PJ (1998) The detection of enzyme activity following sodium dodecyl sulfate-polyacrylamide gel electrophoresis. Anal Biochem 260: 1–17. 9648646 10.1006/abio.1998.2680

[27] Gunne M, Urlacher V (2012) Characterization of the alkaline laccase Ssl1 from *Streptomyces sviceus* with unusual properties discovered by genome mining. PLoS ONE 7: e52360. 23285009 10.1371/journal.pone.0052360 PMC3527528

[28] Matijošytė I, Arends IW, de Vries S, Sheldon RA (2010) Preparation and use of cross-linked enzyme aggregates (CLEAs) of laccases. J Mol Catal B: Enzym 62: 142–148.

[29] D’Souza TM, Boominathan K, Reddy CA (1996) Isolation of laccase gene-specific sequences from white rot and brown rot fungi by PCR. Appl Environ Microbiol 62: 3739–3744. 8837429 10.1128/aem.62.10.3739-3744.1996 PMC168181

[30] Liu Z, Sun X, Qu Y (2008) Cloning Cellobiohydrolase I from *Penicillium decumbens* 114-2 with TAIL-PCR and comparing with its derepressed mutant JU-A10. Acta Microbiol Sin 48: 667–671. 18652301

[31] Tan G, Gao Y, Shi M, Zhang X, He S, et al (2005) SiteFinding-PCR: a simple and efficient PCR method for chromosome walking. Nucleic Acids Res 33: e122. 16077029 10.1093/nar/gni124 PMC1182332

[32] Altschul SF, Gish W, Miller W, Myers EW, Lipman DJ (1990) Basic local alignment search tool. J Mol Biol 215: 403–410. 2231712 10.1016/S0022-2836(05)80360-2

[33] Bendtsen JD, Nielsen H, von Heijne G, Brunak S (2004) Improved prediction of signal peptides: SignalP 3.0. J Mol Biol 340: 783–795. 15223320 10.1016/j.jmb.2004.05.028

[34] Gasteiger E, Hoogland C, Gattiker A, Duvaud S, Wilkins MR, et al.. (2005) Protein identification and analysis tools on the ExPASy server. In: Walker JM, editor. The Proteomics Protocols Handbook. Totowa, NJ: Humana Press. 571–607.

[35] De Castro E, Sigrist CJA, Gattiker A, Bulliard V, Langendijk-Genevaux PS, et al (2006) ScanProsite: detection of PROSITE signature matches and ProRule-associated functional and structural residues in proteins. Nucleic Acids Res 34: W362–W365. 16845026 10.1093/nar/gkl124 PMC1538847

[36] Sievers F, Wilm A, Dineen D, Gibson TJ, Karplus K, et al (2011) Fast, scalable generation of high-quality protein multiple sequence alignments using Clustal Omega. Mol Syst Biol 7: 539. 21988835 10.1038/msb.2011.75 PMC3261699

[37] Tamura K, Peterson D, Peterson N, Stecher G, Nei M, et al (2011) MEGA5: molecular evolutionary genetics analysis using maximum likelihood, evolutionary distance, and maximum parsimony methods. Mol Biol Evol 28: 2731–2739. 21546353 10.1093/molbev/msr121 PMC3203626

[38] Soden DM, Dobson ADW (2003) The use of amplified flanking region-PCR in the isolation of laccase promoter sequences from the edible fungus *Pleurotus sajor-caju* . J Appl Microbiol 95: 553–562. 12911704 10.1046/j.1365-2672.2003.02012.x

[39] Janusz G, Kucharzyk KH, Pawlik A, Staszczak M, Paszczynski AJ (2013) Fungal laccase, manganese peroxidase and lignin peroxidase: Gene expression and regulation. Enzyme Microb Technol 52: 1–12. 23199732 10.1016/j.enzmictec.2012.10.003

[40] Rushmore TH, Morton MR, Pickett CB (1991) The antioxidant responsive element: activation by oxidative stress and identification of the DNA consensus sequence required for functional activity. J Biol Chem 266: 11632–11639. 1646813

[41] Piscitelli A, Giardina P, Lettera V, Pezzella C, Sannia G, et al (2011) Induction and transcriptional regulation of laccases in fungi. Curr Genomics 12: 104–112. 21966248 10.2174/138920211795564331 PMC3129044

[42] Palmieri G, Giardina P, Bianco C, Scaloni A, Capasso A, et al (1997) A novel white laccase from *Pleurotus ostreatus* . J Biol Chem 272: 31301–31307. 9395457 10.1074/jbc.272.50.31301

[43] Liu W, Chao Y, Liu S, Bao H, Qian S (2003) Molecular cloning and characterization of a laccase gene from the basidiomycete *Fome lignosus* and expression in *Pichia pastoris* . Appl Microbiol Biotechnol 63: 174–181. 12898062 10.1007/s00253-003-1398-0

[44] Eggert C, LaFayette PR, Temp U, Eriksson K-EL, Dean JFD (1998) Molecular analysis of a laccase gene from the white rot fungus *Pycnoporus cinnabarinus* . Appl Environ Microbiol 64: 1766–1772. 9572949 10.1128/aem.64.5.1766-1772.1998 PMC106228

[45] Xu F, Berka RM, Wahleithner JA, Nelson BA, Shuster JR, et al (1998) Site-directed mutations in fungal laccase: effect on redox potential, activity and pH profile. Biochem J 334: 63–70. 9693103 10.1042/bj3340063 PMC1219662

[46] Fan F, Zhuo R, Sun S, Wan X, Jiang M, et al (2011) Cloning and functional analysis of a new laccase gene from *Trametes* sp. 48424 which had the high yield of laccase and strong ability for decolorizing different dyes. Bioresour Technol 102: 3126–3137. 21094600 10.1016/j.biortech.2010.10.079

[47] Nozaki K, Beh CH, Mizuno M, Isobe T, Shiroishi M, et al (2008) Screening and investigation of dye decolorization activities of basidiomycetes. J Biosci Bioeng 105: 69–72. 18295724 10.1263/jbb.105.69

