# Supplementary material for: Purification and Characterization of a Novel Laccase from Cerrena sp. HYB07 with Dye Decolorizing Ability
Source: PLoS One. 2014 Oct 30;9(10):e110834. doi: 10.1371/journal.pone.0110834 (PMC4214704; doi:10.1371/journal.pone.0110834)
Supplement: Figure S1 — The result of the MALDI-TOF MS/MS analysis of the purified LacA protein. (DOCX) [file pone.0110834.s001.docx]

**Supplementary Data**

Fig. S1 The result of the MALDI-TOF MS/MS analysis of the purified LacA protein.

LacA was identified to be the protein product encoded by the *LacA* gene.

(A) Peptide mass fingerprint of the tryptic digest of LacA. * indicates unique peptides further identified by MS/MS.

(B) MS/MS profile of the peptide with a mass of 1617.79 Da (SAGQTTPNYVDPIVR).

(C) MS/MS profile of the peptide with a mass of 1950.96 Da (YSAVLNANQPVGNYWVR).

| A | 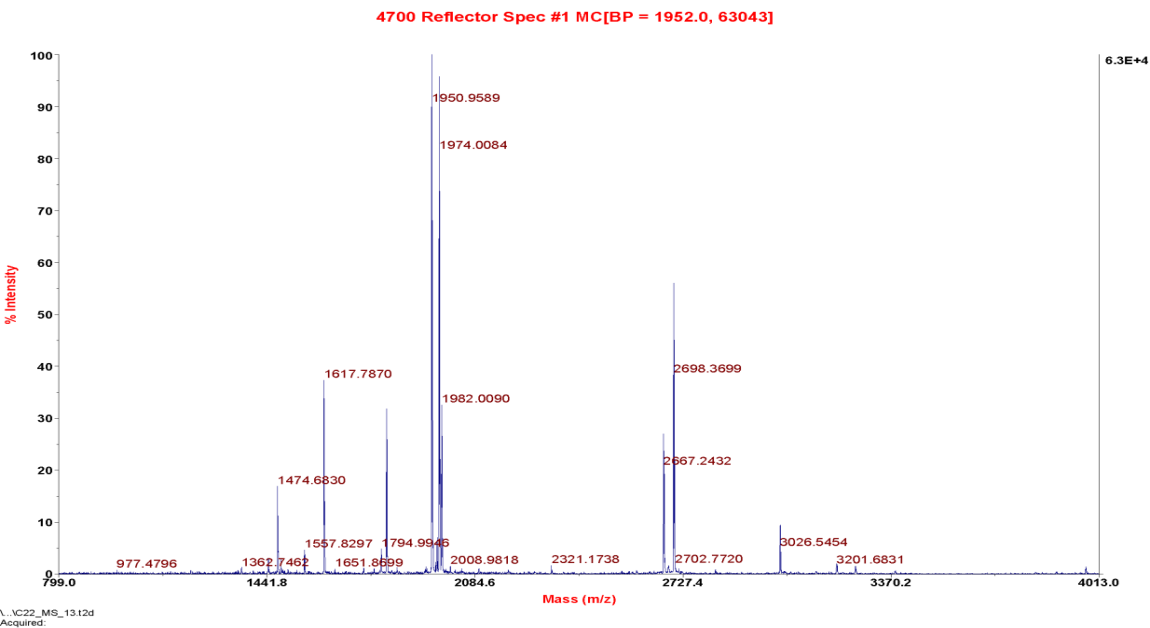 *  * | | |
| --- | --- | --- | --- |
| B | 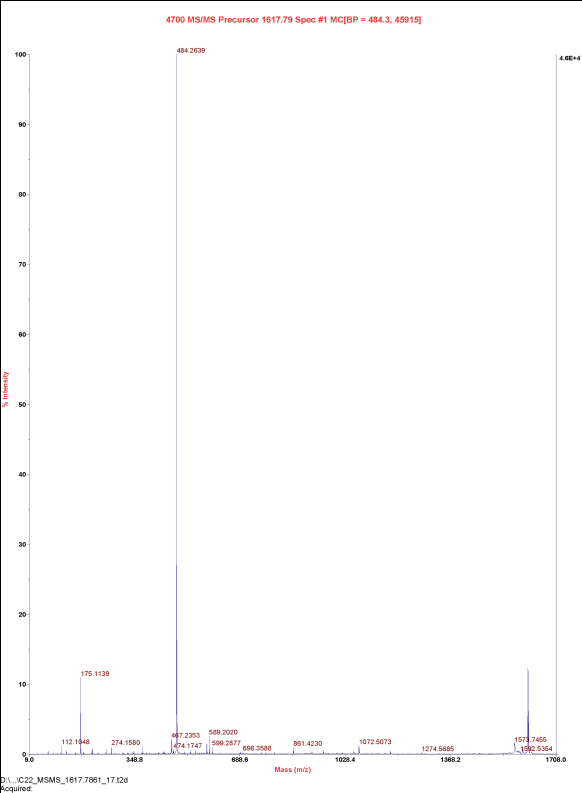 | C | 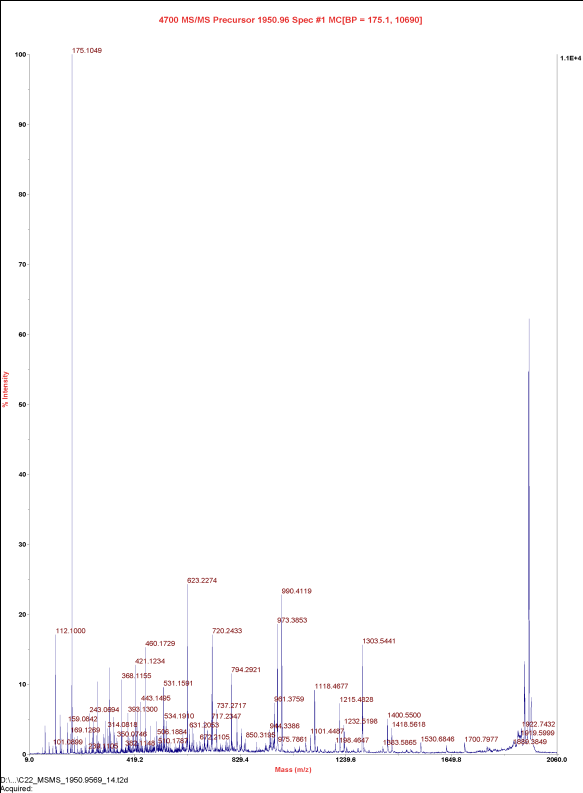 |
